# The Doctor's Vade-Mecum

**Published:** 1917-05-12

**Authors:** 


### THE DOCTOR'S VADE-MECUM.

### Simple Rules for Medical Practitioners.

A FUNNY CONTRIBUTION : BY A STUDENT OF THOMAS LOVE PEACOCK'S SCHOOL.

THIS little article is intended to assist the budding doctor in his profession. All the information herein brought together could be acquired by a four years' course at any of our big hospitals. It is, in fact, usually acquired in that way. But in case the student should prefer to have a good time during those golden years he can cut all the more exhausting lectures and rely with perfect safety on these few simple rules. Provided the following hints are carefully followed, any doctor well under the average of ability can be sure of a first-rate reputation.

1. *Treatment of Indigestion.*—Doctors really know nothing whatever about indigestion, its cause and cure. Many of them suffer terribly from it themselves and die dyspeptic deaths. They have to grin and bear it or bear it without grinning, whichever they prefer.

But, of course, when summoned to attend a patient afflicted in this way, you must *know*. Your safest plan will be to rely on the tea-and-coffee stunt. Ask the dyspeptic if he takes tea. Should he say "Yes," pull a long face (unless you have a long face naturally) and say, "Ah, I thought so. You mustn't touch a drop of tea. Take coffee. Tea—for you—is a poison." Should he say "No," it's a sure thing he drinks coffee. There is your chance. Tell him that coffee—for him—is little better than arsenic. He must take tea. By this method you can secure a good reputation for knowing something about indigestion.

2. *Diagnosis.*—This is one of the most difficult tasks that will ever confront you in your professional capacity. Many of the tip-top specialists absolutely refuse to diagnose. They prefer to put their patients into nursing homes and leave them there until a simple epidemic comes along. They can then trace all the trouble to suppressed measles or whooping-cough. But as a young man with a reputation to make, you ought to try to diagnose. The laws of chance (all doctors know there is such a thing as chance) will sometimes come to your aid. You may even score a "bull" occasionally. In cases where you really haven't the slightest notion what is the matter, proceed to give the patient a list of the diseases he's not got. You can feel him for appendicitis and inform him it's not that. Look critically at any spots he may have and assure him it's not smallpox. Feel his glands and tell him cheerily, "Well, anyway, it isn't mumps." Listen to his heart and lungs and negative his suggestion that it might be influenza. The mere idea of having escaped so many ailments will do him good. His spirits will imperceptibly rise and his temperature (if he has one) will imperceptibly fall. Also he will look upon you as a most painstaking practitioner and you will go up 50 per cent. in his estimation.

3. *Need for Warmth.*—Often a disease will not come out. You may know perfectly well what the patient is suffering from and yet be doomed day after day to watch in vain, as a cat watches a deserted mousehole. When this happens, there is only one thing to do. You must get him into a sudden heat. It doesn't matter in the least how you do it. But do it you must. Flatly contradict him on some subject on which you know he is better informed than yourself. Be rude to him. Tell him his windows want cleaning and that it's high time he had the room re-papered. Only in this way will the requisite degree of heat be produced. To strike heat out of a naturally quick temper is far the most direct method.

4. *Absence of Pain.*—Should the patient admit that he is not in any pain, lose no time in rectifying this. To send for you at all, if he is not in pain, is little short of an insult. You did not receive an expensive education to be flouted and made a fool of. To *be* a fool is one thing : to be *made* a fool of quite another. In the former case you can't help it, but in the latter emphatically you can. There are plenty of good mixtures that will produce a splitting headache in a very short time. Send round one of these, having first questioned him narrowly about his head. Infer that, according to all the rules, he *ought* to have a pain there. You may even go so far as to hint that unless severe headache supervenes he may be in considerable danger. In this way you will have your revenge for having been fooled and add considerably to your reputation. The headache, when it comes, will give him confidence in you. He will feel instinctively that you know what you are doing, though *he* may not.

5. *Rescues from the Grave.*—It is imperative that every member of the patient's household shall know at once what a difficult case you have been called in to attend. Start by hinting that you ought to have been sent for at least two days ago, and if the patient be a Roman Catholic tell them to send immediately for the priest. See him yourself and advise him to administer extreme unction in its most extreme form. He's bound to take your word for it that the need is urgent. You can also rub it in (in strict confidence) that from certain unmistakable symptoms it's only too apparent the patient has been no better than he should be. This will assure priestly dispatch. Then, during the slow process of recovery (never let these cases improve too speedily), you will win great glory. To have rescued a patient from the brink of the grave is a feat worth talking about. Talk about it.

6. *The Shock Cure.*—If you think a shock will do the patient good (many diseases, including deafness, blindness, madness and sadness or hypochondriasis yield to shock), choose a cold, raw morning and drive your car, bare-handed, to the patient's house. Go straight up to his room, shout him a breezy greeting, brusquely fling off the bed-clothes and put your cold hands suddenly on his warm stomach under the pretext of feeling for his appendix. This will give a first-class shock, and if it doesn't cure him, will at least break down that professional reserve of which so many patients complain.

